# *In vivo* gene delivery and expression by bacteriophage lambda vectors

**DOI:** 10.1111/j.1365-2672.2006.03182.x

**Published:** 2007-05

**Authors:** HA Lankes, CN Zanghi, K Santos, C Capella, CMP Duke, S Dewhurst

**Affiliations:** 1 Department of Microbiology and Immunology, University of Rochester Medical Center Rochester, NY, USA; 2 Cancer Center, University of Rochester Medical Center Rochester, NY, USA

**Keywords:** bacteriophage, gene expression, gene transfer, immunization, luciferase, phage lambda, vaccine

## Abstract

**Aims:**

Bacteriophage vectors have potential as gene transfer and vaccine delivery vectors because of their low cost, safety and physical stability. However, little is known concerning phage-mediated gene transfer in mammalian hosts. We therefore performed experiments to examine phage-mediated gene transfer *in vivo*.

**Methods and Results:**

Mice were inoculated with recombinant lambda phage containing a mammalian expression cassette encoding firefly luciferase (luc). Efficient, dose-dependent *in vivo* luc expression was detected, which peaked within 24 h of delivery and declined to undetectable levels within a week. Display of an integrin-binding peptide increased cellular internalization of phage *in vitro* and enhanced phage-mediated gene transfer *in vivo*. Finally, *in vivo* depletion of phagocytic cells using clodronate liposomes had only a minor effect on the efficiency of phage-mediated gene transfer.

**Conclusions:**

Unmodified lambda phage particles are capable of transducing mammalian cells *in vivo*, and may be taken up – at least in part – by nonphagocytic mechanisms. Surface modifications that enhance phage uptake result in more efficient *in vivo* gene transfer.

**Significance and Impact of the Study:**

These experiments shed light on the mechanisms involved in phage-mediated gene transfer *in vivo*, and suggest new approaches that may enhance the efficiency of this process.

## Introduction

Bacteriophage vectors have potential as vaccine delivery and gene transfer vectors because of their genetic tractability, inexpensive production and suitability for large-scale production (Chen and Lim 1996), as well as their physical stability and compatibility with simple storage and formulation methods such as desiccation (Jepson and March 2004). In addition, phages have been experimentally administered to animals and safely used in humans for applications that include the treatment of bacterial infections (reviewed in Barrow and Soothill 1997; Stone 2002), and more recently, recombinant phage coat proteins, which form virus-like particles, have also been used for immunization (Maurer *et al.* 2005).

Larocca *et al.* (1998,1999) have shown that filamentous phage vectors displaying host-derived ligands on their surface can undergo receptor-mediated endocytosis, resulting in the expression of phage-encoded genes in mammalian cells. Similarly, lambda phage can be targeted to mammalian cells by surface display of appropriate proteins or peptides (Dunn 1996).

March and colleagues have shown that immunization of mice and rabbits with unmodified lambda phage particles encoding the hepatitis B surface antigen (HBsAg) or antigens derived from *Mycoplasma mycoides* under control of the cytomegalovirus (CMV) promoter results in a strong antigen-specific humoural immune response (Clark and March 2004; March *et al.* 2004,^2006^; ). These results indicate that even unmodified, nontargeted lambda phage particles can mediate *in vivo* gene delivery, possibly because of internalization by antigen-presenting cells (APCs) (Clark and March 2004).

In this article, we present a number of *in vivo* findings pertinent to the future development of lambda as both a vaccine delivery vector and a gene transfer vector. In the experiments presented, we used a *trans*-complementation system to produce lambda phage particles bearing different versions of the gpD coat protein (Zanghi *et al.* 2005). These particles contained a modified lambda phage genome that incorporates a CMV promoter-driven mammalian luciferase (luc) reporter gene cassette (Eguchi *et al.* 2001; Zanghi *et al.* 2005). We then used these phage particles to characterize phage-mediated gene transfer *in vivo*, using a real-time imaging system. These studies provide proof-of-concept support for the notion that directed targeting of lambda phage vectors, through surface protein modification, may result in more efficient gene transfer *in vivo*. This study also suggests that phage vectors may be taken up, at least in part, via nonphagocytic mechanisms, possibly including macropinocytosis. These findings offer important insight into the basis for phage-mediated gene transfer and suggest possible modifications for improving phage-based vaccine delivery systems.

## Materials and methods

### LambdaD1180 (luc) lysogens

LambdaD1180 (no luc) and lambdaD1180 (luc) lysogens (*Dam15 del EcoRI-SacI cIts857 nin5 Sam100*) were provided by Dr Mahito Nakanishi (Eguchi *et al.* 2001). LambdaD1180 (luc) lysogens contain the firefly luc gene, under the transcriptional control of the CMV immediate early promoter; lambdaD1180 (no luc) lysogens contain no insert.

### pTrc-gpD expression plasmids

The pTrc-gpD, pTrc-CDF-gpD and pTrc-gpD-3JCLI4 expression plasmids have been described (Zanghi *et al.* 2005). The pTrc-gpD-3JCLI4 plasmid encodes gpD, fused to a high-affinity *α*_v_*β*_3_ binding protein (3JCLI4) (Richards *et al.* 2003; Zanghi *et al.* 2005).

### Lambda phage preparation

Lysogens of *Escherichia coli* TOP10 cells (Invitrogen, Carlsbad, CA, USA) containing lambdaD1180 (no luc) or lambdaD1180 (luc) were transformed with the pTrc-gpD plasmid containing wild-type gpD. Additionally, lambdaD1180 (luc) lysogens were cotransformed with the pTrc-gpD-3JCLI4 and gpD-CDF-gpD plasmids (Zanghi *et al.* 2005). Lysogens were induced as described, and phages were purified by CsCl density gradient centrifugation, prior to titrating on LE392 *E. coli* cells and immunoblot analysis (to verify the presence of the expected recombinant forms of gpD) (Zanghi *et al.* 2005).

LambdaD1180 (luc) lysogens were also prepared via a lytic method to produce phage complemented with phage-encoded gpD. In this case, phages prepared lytically were grown in *E. coli* host cells that contained an amber suppressor tRNA and thus could produce a functional gpD coat protein from the amber-mutated gpD gene contained in the phage genome. To do this, LE392 *E. coli* cells (*e14*-(*McrA-*) *hsdR514 supE44 supF58 lacY1* or ΔΔ(*lacIZY*)*6 galK2 galT22 metB1 trpR55*) grown in NZCYM plus 0·2% maltose were infected with gpD (luc) phage and grown at 37°C until lysis was observed. Upon lysis, chloroform (Sigma, St Louis, MO, USA) was added to lyse the remaining intact host cells and DNase I (Worthington Biochemical Corp., Freehold, NJ, USA) was added to a final concentration of 1 *μ*g ml^−1^ to remove contaminating nucleic acids. NaCl was added to a final concentration of 1 mol l^−1^ and lysates were cleared by centrifugation. Phage was precipitated by the addition of polyethylene glycol-8000 (PEG; Sigma-Aldrich) to a final concentration of 10% (w/v). Precipitated phage particles were pelleted by centrifugation, resuspended in suspension media (SM; 100 mmol l^−1^ NaCl, 10 mmol l^−1^ MgSO4·7H2O, 50 mmol l^−1^ Tris–HCl (pH 7·5), 0·1% gelatin), extracted with chloroform and purified by CsCl density gradient ultracentrifugation. Purified phages were dialysed against 10 mmol l^−1^ NaCl, 50 mmol l^−1^ Tris–HCl (pH 8·0), 10 mmol l^−1^ MgCl2, prior to titrating on LE392 *E. coli* cells.

### Mice

BALB/c mice were obtained from Taconic Laboratories (Hudson, NY, USA) and maintained according to University of Rochester and NIH guidelines. Mice were injected intradermally (ID), via the tail base, intramuscularly (IM), in the thigh, or via the intraperitoneal (IP) route, using a 28G, 0·5 inches, 0·5 ml insulin syringe (BD Biosciences, San Jose, CA, USA).

### In vivo imaging of luciferase expression

Mice were injected with CsCl-purified lambda phage, purified lambda luc DNA, a luc reporter plasmid (gWiz; Aldevron, Fargo, ND, USA), in 50 or 100 *μ*l total volume. Luc expression was detected using a Xenogen IVIS system (Hopkinton, MA, USA) to image *in vivo* bioluminescence in real time (Fan *et al.* 2005); luc expression was confined to the local site of injection in all cases.

### Analysis of luciferase expression in tissue lysates

Mice were killed following imaging and 6 mm tail base tissue samples (from the local site of phage injection) were collected using a tissue punch. Tissue samples were added to 1× passive lysis buffer (Promega, Madison, WI, USA) and cryopreserved at −80°C. Prior to homogenate preparation, tissues were thawed, flash frozen in liquid nitrogen, pulverized, resuspended in 1× passive lysis buffer and homogenized (Ultrathurrax, Ika-Werke, Germany). Protein concentrations were determined by Bradford assay (Bio-Rad, Hercules, CA, USA). Luc activity was determined as described (Fan *et al.* 2005). Prior to the analysis, homogenates were thawed and 25 *μ*l of the lysate (0·5 *μ*g *μ*l^−1^) was combined with 25 *μ*l of firefly luc substrate (Promega). Light emission was measured in a white 96-well plate using the SpectraCount version 3.0 luminometer (Packard; Perkin Elmer, Boston, MA, USA).

### DNase I treatment

DNase I (10 U; New England Biolabs, Ipswich, MA, USA) or DNase I buffer (10 mmol l^−1^ Tris–HCl, 2 mmol l^−1^ CaCl_2_, 100 *μ*g/ml BSA, 50% glycerol) was added to 1 × 10^11^ PFU of gpD (luc) bacteriophage or 1 × 10^11^ PFU of gpD (no luc) physically mixed with 5 *μ*g of lambda luc DNA in a total volume of 50 *μ*l of SM. After incubation at 37°C for 30 min, DNase I or DNase I buffer-treated (mock) phage or phage plus DNA was injected into mice and luc expression was imaged 24 h later.

### Pre-immunization with bacteriophage lambda

Mice were pre-immunized via IM injection with either 1 × 10^11^ PFU of gpD (no luc) in 50 *μ*l total volume, or 50 *μ*l of SM alone. Immediately prior to immunization, serum was collected. Two weeks following immunization, serum was again collected and mice were then injected ID with 1 × 10^11^ PFU of gpD (luc) phage in 50 *μ*l total volume. Twenty-four hours later, luc expression was imaged.

### ELISA detection of bacteriophage lambda antibodies from sera

Sera collected were tested by ELISA for the presence of antibodies directed against bacteriophage lambda. Microtitre plates were coated overnight at 4°C with 1 × 10^9^ PFU of wild-type bacteriophage lambda (strain W60; ATCC) diluted in 1× phosphate-buffered saline (PBS). After blocking with 3% BSA in 1× PBS, sera were added to wells in serial fourfold dilutions in 3% BSA in 1× PBS. Wells were washed and horseradish-peroxidase-conjugated anti-mouse IgG (Fc specific; Sigma) was added at a dilution of 1:70 000 in 3% BSA in 1× PBS. Wells were developed with Sure Blue substrate (KPL, Gaithersburg, MD, USA) and read at an absorbance of 450 nm. It is noted that this IgG-specific ELISA may underestimate antibody titres to a single phage vaccination, as it will fail to detect other antibody classes (such as IgM).

### In vitro internalization assay

The K562-*α*_v_*β*_3_ cells (expressing *α*_v_*β*_3_ integrin on their surface) were provided by Dr Scott Blystone (SUNY Upstate Medical University, Syracuse, NY, USA) and were incubated in 96-well plates with various concentrations of purified 3JCLI4 protein (provided by Casey Maguire) for 15 min at 4°C prior to the addition of phage. Either gpD (luc) or 3JCLI4 (luc) phage was added to cells at a multiplicity of infection (MOI) of 1 × 10^5^, centrifuged for 15 min and incubated at 37°C for an additional 1 h and 45 min. Following incubation, cells were washed with an ice-cold acid wash (0·3 mol l^−1^ acetic acid, 0·5 mol l^−1^ NaCl, pH 2·5) and manually lysed by passing through a 28G, 0·5 inches, 0·5 ml insulin syringe. This results in >99% lysis of cells, as measured by trypan blue dye exclusion. Internalized phages in the cell lysates were then quantified by titration on LE392 *E. coli* cells. It is noted that this assay almost certainly underestimates phage internalization as it counts only those phages that remain intact and infectious for *E. coli* (and does not count phage particles that may be fully or partially degraded).

### In vitro analysis of luc expression

The K562-*α*_v_*β*_3_ cells and control K562 cells were seeded in 96-well plates. Either gpD (luc) or 3JCLI4 (luc) phage was then added to cells at MOI of 1 × 10^5^, and the plates were subjected to centrifugation at 900 *g* for 15 min, in order to enhance the efficiency of phage binding to the target cells (O'Doherty *et al.* 2000; Scanlan *et al.* 2005; Harui *et al.* 2006a). After this, the cultures were returned to a 37°C incubator for an additional 1 h and 45 min. Following incubation, phage-containing media were removed, cells were washed once and incubated in complete media to allow for luc expression. Forty-eight hours later, 1× passive lysis buffer was added and cells were subjected to two freeze/thaw cycles to ensure cell lysis. Protein concentrations from cell lysates were normalized using a Bradford assay. Luc activity was determined as described (Fan *et al.* 2005). Prior to analysis, 20 *μ*l of the lysate was combined with 100 *μ*l of firefly luc substrate. Light emission was measured in a white 96-well plate using the SpectraCount version 3.0 luminometer.

### Phage stability assays

The phages were suspended in 10% normal mouse serum (diluted in SM), at a final concentration of approx. 2 × 10^10^ PFU ml^−1^ and were then incubated for 30 min at 37°C prior to titrating on LE392 *E. coli* cells. Similar experiments were conducted by incubating phage with various concentrations of EDTA and SDS for 30 min at room temperature prior to titrating on LE392 *E. coli* cells.

### Macrophage depletion via clodronate liposomes

Clodronate was purchased from Sigma and clodronate liposomes were generated using methods developed by van Rooijen *et al.* (van Rooijen and van Nieuwmegen 1984; van Rooijen *et al.* 1989; van Rooijen and van Kesteren-Hendrikx 2002). BALB/c mice were injected with clodronate liposomes via a combined ID (100 *μ*l) and IP (200 *μ*l) route. Forty-eight hours following clodronate liposome injection, mice were injected ID with phage or DNA, and IVIS imaging was performed 24 h thereafter. Mice were then killed and splenocytes were subjected to flow cytometric analysis using monoclonal antibodies specific for the myeloid marker F4/80 (antibody provided by Dr Edith Lord).

## Results

### In vivo luciferase expression in BALB/c mice

**Figure 1:**
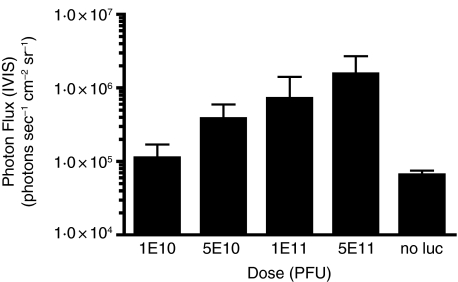
*In vivo* luciferase (luc) expression in BALB/c mice is dose dependent. Mice (three per group) were injected ID at the tail base with 1 × 10^10^, 5 × 10^10^, 1 × 10^11^ or 5 × 10^11^ PFU of gpD (luc) phage or 1 × 10^11^ PFU of gpD (no luc) phage. Twenty-four hours later, luc expression was imaged at the tail base site of injection. The graph is representative of the average photon flux (photons sec^−1^ cm^−2^ sr^−1^) and SD for each group.

To examine the mechanism(s) involved in *in vivo* gene expression from bacteriophage lambda vectors, a phage containing a firefly luc expression cassette was used to perform real-time analysis of luc expression *in vivo*. We first performed a dose–response analysis, in which mice were injected ID, via the tail base, with 1 × 10^10^, 5 × 10^10^, 1 × 10^11^, or 5 × 10^11^ PFU of gpD (luc) phage or 1 × 10^11^ PFU of gpD (no luc) phage, in 50 *μ*l total volume. Twenty-four hours later, luc expression was measured. *In vivo* luc gene expression was found to correlate with phage dose (Fig. 1) and to be localized to the local site of vector delivery at the tail base (Supplementary data, Fig. 1S). On the basis of these results, a dose of 1 × 10^11^ PFU was chosen for use in follow-up experiments.

### Time course analysis of phage-mediated gene expression

**Figure 2:**
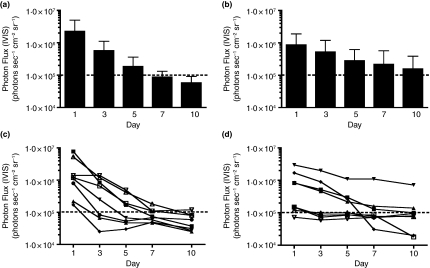
*In vivo* luciferase (luc) expression in BALB/c mice injected with wild-type luc-encoding phage persists over a time frame very similar to that of mice injected with purified lambda luc DNA. Mice (eight per group) were injected ID at the tail base with either 1 × 10^11^ PFU of wild-type gpD (luc) phage (panels a, c) or 5 *μ*g of purified lambda luc DNA (panels b, d); note that 5 *μ*g DNA corresponds to the expected amount of genomic lambda DNA contained in 1 × 10^11^ PFU of lambda phage. Luc expression was measured at the tail base site of injection, 1, 3, 5, 7, and 10 days following injection. Results shown in panels a and b represent mean luc expression values ± SDs. *In vivo* luc expression in mice injected with gpD (luc) is similar to expression levels obtained from mice injected with 5 *μ*g purified lambda luc DNA. Panels c and d represent an analysis of luc expression in each of the individual eight animals from each experimental group that is represented in the summary graphs (panels a and b). The dashed horizontal line drawn across each of the panels denotes the cut-off of the assay (1 × 10^5^ photons sec^−1^ cm^−2^ sr^−1^).

The time course of phage-mediated gene expression was analysed, following ID delivery of either gpD (luc) particles or purified genomic lambda luc DNA alone (Fig. 2). In this experiment, 5 *μ*g of purified genomic lambda luc DNA was used, as this corresponds to the expected amount of genomic lambda DNA contained in 1 × 10^11^ PFU of lambda phage particles. Luc expression peaked at the 24 h time point in both cases, and declined to undetectable levels within 7 days of injection of phage particles. Expression from the purified lambda luc DNA alone showed a trend towards a more prolonged duration, but this result did not achieve statistical significance.

### Treatment of purified phage with DNase I does not affect in vivo luciferase expression

**Figure 3:**
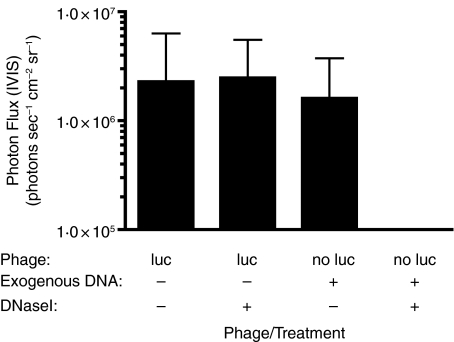
Luciferase (luc)-encoding phage is resistant to DNase I treatment. Mice (eight per group) were injected ID at the tail base with 1 × 10^11^ PFU of gpD (luc) phage that were either treated with 10 U of DNase I for 30 min at 37°C prior to injection, or not exposed to DNase I. As a control, mice (four per group) were injected with 1 × 10^11^ PFU of matching phage that lacked the luc expression cassette (no luc), which were physically mixed with 5 *μ*g of lambda luc DNA and then either treated with 10 U of DNase I for 30 min at 37°C prior to injection, or not exposed to DNase I. Twenty-four hours later, luc expression was measured at the tail base site of injection. DNase I treatment had no detectable effect on luc expression levels in mice that were injected with gpD (luc) phage (*P* > 0·05, Student's two-tailed *t*-test). However, the amount of DNase I added was sufficient to degrade 5 *μ*g of exogenous DNA (compare luc expression levels in the two no luc groups).

Jepson and March (2004) have demonstrated that lambda phage particles represent a highly stable DNA transfer system. This finding suggests that phage-encapsidated DNA is protected from environmental damage, but can be released within mammalian cells, following uptake of phage particles. This prediction was experimentally tested, by treating CsCl-gradient purified lambda phage particles encoding a luc reporter gene with DNase I and then administering the treated phage particles to mice. As shown in Fig. 3, DNase I treatment of gpD (luc) phage had no effect on *in vivo* luc expression. The DNase I treatment was sufficient to degrade 5 *μ*g of exogenous lambda luc DNA in the presence of 1 × 10^11^ PFU of phage and eliminate luc signal in this group.

### In vivo luc expression in BALB/c mice injected with gpD (luc) phage is increased when mice are pre-immunized with bacteriophage lambda

The human intestinal tract is colonized by commensal microbial flora that includes *E. coli*. This, combined with the isolation of bacteriophages from raw sewage and faecal samples (Schluederberg *et al.* 1980), suggests that some humans may harbour enteric coliphages. Consistent with this, coliphage neutralizing activity has been detected in normal human serum (Cowan 1962).

**Figure 4:**
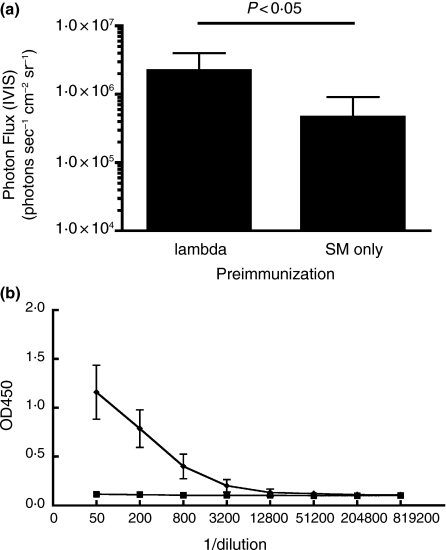
*In vivo* luciferase (luc) expression in BALB/c mice injected with gpD (luc) phage is increased when mice are pre-immunized with bacteriophage lambda. Mice were immunized IM with either 1 × 10^11^ PFU of gpD (no luc) bacteriophage lambda (lambda) in 50 *μ*l of suspension media or 50 *μ*l of suspension media alone (SM only). Two weeks postimmunization, all mice were injected ID at the tail base with 1 × 10^11^ PFU of gpD (luc) phage, and luc expression was then measured 24 h later at the tail base site of injection. (a) Data shown represent mean luc expression values ± SDs; the data shown were combined from two separate experiments that used a total of seven mice. There was a statistically significant difference in *in vivo* luc gene expression between mice that were pre-immunized with bacteriophage lambda *vs* mice that were pre-immunized with suspension media alone (*P* < 0·05, Student's two-tailed *t*-test). (b) Sera were collected from mice at 14 days following the initial phage immunization and analysed for lambda-specific IgG antibodies by ELISA. Antibodies specific for bacteriophage lambda were detected in mice pre-immunized with bacteriophage lambda, but not in control mice.

While the seroprevalence of lambda phage-specific antibodies within human populations is not known, such antibodies might represent a barrier to the use of phage lambda in humans. An experiment was therefore conducted to determine the impact of pre-existing immunity to bacteriophage lambda on the efficiency of lambda-vectored reporter gene expression *in vivo*. To do this, mice were pre-immunized with wild-type bacteriophage lambda. Two weeks later, mice were injected ID with 1 × 10^11^ PFU of gpD (luc) phage. A statistically significant increase in luc expression was detected in the pre-immunized mice (which had high titres of phage-specific antibodies), when compared to control mice (Fig. 4). Thus, pre-existing immunity to bacteriophage lambda failed to neutralize locally delivered phage particles, and in fact, had an enhancing effect on vector-mediated gene transfer.

### 3JCLI4 (luc) phage targets α _v_β _3_ integrin in vitro and increases in vivo luciferase expression

We have previously described the construction of luc-encoding phage vectors in which the major phage coat protein (gpD) is fused to an *α*_v_*β*_3_ integrin-binding peptide (3JCLI4) (Richards *et al.* 2003). This peptide binds with high affinity and specificity to *α*_v_*β*_3_, but not to other integrins (Richards *et al.* 2003).

We sought to determine the ability of this targeted phage to specifically internalize into mammalian cells expressing *α*_v_*β*_3_*in vitro*. We elected to target this integrin for two major reasons. First, this receptor is known to play a role in the binding and/or internalization of a number of mammalian viruses – including adenoviruses (Wickham *et al.* 1993; Mathias *et al.* 1994; Huang *et al.* 1995) and some hantaviruses (Gavrilovskaya *et al.* 1998; Song *et al.* 2005). Secondly, the receptor has been successfully used to enhance the targeting of modified virus vectors to dendritic cells (Asada-Mikami *et al.* 2001; Okada *et al.* 2001; Harui *et al.* 2006b; Maguire *et al.* 2006), which are known to express the receptor (Rubartelli *et al.* 1997).

**Figure 5:**
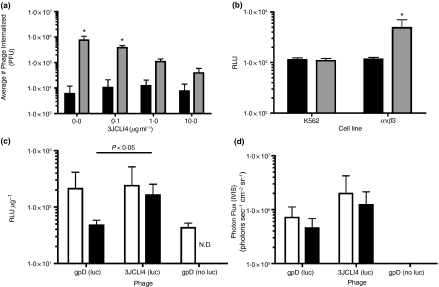
3JCLI4 (luc) phage targets *α*v*β*3 *in vitro* and increases *in vivo* luc expression. (a) K562-*α*v*β*3 cells were incubated with gpD (luc) (▪) or 3JCLI4 (luc) (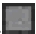) phage (containing a modified gpD protein bearing the 3JCLI4 integrin-binding peptide) at an MOI of 10^5^, in the presence or absence of increasing concentrations of soluble 3JCLI4 protein. Two hours later, cells were washed and lysates were prepared in order to quantify internalized phage, which were then titrated on LE392 *Escherichia coli* cells. Data shown represent mean phage titres ± SDs (calculated from three independently analysed wells of a cell culture dish, each of which was titrated in triplicate). There was a statistically significant increase in phage internalization between the 3JCLI4 (luc) phage particles and the gpD (luc) phage particles (*P < 0·05, two-way anova, Tukey's post-test). In addition, internalization of the 3JCLI4 (luc) phage was reduced, by the addition of soluble 3JCLI4 protein, in a dose-dependent fashion (this reduction was statistically significant at all concentrations of soluble 3JCLI4 that were added; *P* < 0·001 in all cases, two-way anova, Tukey's post-test). (b) gpD (luc) (▪) or 3JCLI4 (luc) (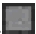) phage was added to K562-*α*v*β*3 or wild-type K562 cells in 96-well plates, at an MOI of 10^5^. The plates were subjected to centrifugation at 900 *g* for 15 min, in order to enhance the efficiency of phage binding to the target cells (O'Doherty *et al.* 2000; Scanlan *et al.* 2005; Harui *et al.* 2006a). After this, the cultures were returned to a 37°C incubator for 1 h and 45 min, and the cells were then washed to remove unbound phage. The cultures were again returned to a 37°C incubator, and 48 h later, cell lysates were prepared. After normalization of the protein content of the cell lysates, luc expression was measured. Data shown represent mean luc expression values ± SDs (calculated from three independent experiments, each of which analysed triplicate wells of a cell culture dish). There was a statistically significant increase in luc expression between the 3JCLI4 (luc) phage particles and the gpD (luc) phage particles, when tested in K562-*α*v*β*3 cells but not when tested in wild-type K562 cells (*P < 0·05, two-way anova, Tukey's post-test). Thus, 3JCLI4 (luc) can be targeted to cells expressing *α*v*β*3. (c, d) To examine the ability of 3JCLI4 (luc) to increase *in* vivo gene delivery, mice (eight per group) were injected ID at the tail base with 1 × 10^11^ PFU of either gpD (luc) phage or 3JCLI4 (luc) phage (containing a modified gpD protein bearing the 3JCLI4 integrin-binding peptide). As a negative control, four mice were injected with 1 × 10^11^ PFU of gpD (no luc). (c) Mice were killed at either 1 or 3 days following phage injection, and the tail base site of injection was excised using a tissue punch. The tissue sample was then homogenized in luc sample buffer, and luc activity was measured using a chemiluminescent assay; results are expressed as relative light units (RLU) per *μ*g of tissue extract. Data shown represent mean luc expression values ± SDs (four mice per group). There was a statistically significant difference in *in vivo* luc gene expression between mice that received 3JCLI4 (luc) phage particles *vs* animals that received the gpD (luc) phage particles at the 3-day time point (*P* < 0·05, Student's two-tailed *t*-test). The data for the 1-day time point did not achieve statistical significance. In this experiment, the control group (which received ‘no luc’ phage) was analysed at 1 day following phage delivery (only). The analysis of control animals was not repeated at the 72-h time point (N.D.), because it was felt that a single time point was sufficient to establish the background in the luc assay (□, 24 h; ▪, 72 h). (d) Luc expression was analysed at the tail base site of injection in live animals at the day 1 and 3 time points, using the Xenogen IVIS system. Data shown represent mean luc expression values ± SDs (eight mice per group at day 1 and four mice per group at day 3). *In vivo* luc gene expression was found to be greater in animals injected with 3JCLI4 (luc), although this increase did not achieve statistical significance (*P* > 0·05, Student's two-tailed *t*-test). In this experiment, the control group (which received ‘no luc’ phage) was analysed at both 1 and 3 days following phage delivery. The luc signal measured in these control animals fell below the background cut-off of the assay (□, 24 h; ▪, 72 h).

Wild-type gpD (luc) or 3JCLI4 (luc) phage was incubated with K562-*α*_v_*β*_3_ cells in the presence or absence of various concentrations of competing, soluble 3JCLI4 protein. Following an acid wash to remove extracellular phages, cell lysates were prepared, and internalized phage were titrated on LE392 *E. coli* cells. 3JCLI4 (luc) showed increased internalization *vs* gpD (luc) phage. 3JCLI4 (luc) internalization was reduced by the addition of soluble, competitor 3JCLI4 protein, in a dose-dependent fashion, indicating that internalization occurred in a receptor-specific manner (Fig. 5a).

This result was confirmed by an analysis of phage-mediated gene transfer in parental K562 cells and K562-*α*_v_*β*_3_ cells, following spinoculation with wild-type gpD (luc) or 3JCLI4 (luc) (O'Doherty *et al.* 2000; Scanlan *et al.* 2005; Harui *et al.* 2006a). This experiment revealed that the 3JCLI4 (luc) phage elicited a higher level of luc expression in K562-*α*_v_*β*_3_ cells when compared with parental K562 cells, whereas no such enhancement was detected when cells were incubated with the wild-type gpD (luc) phage (Fig. 5b). Collectively, these *in vitro* data show that 3JCLI4 (luc) can be targeted to cells expressing *α*_v_*β*_3_.

We next examined the effect of vector targeting on luc gene expression *in vivo*. BALB/c mice were injected ID with 1 × 10^11^ PFU of either wild-type gpD (luc) phage or 3JCLI4-targeted (luc) phage. A control group of animals received gpD (no luc) phage lacking the luc insert. Luc expression was then examined by two independent assays at 1 and 3 days following phage inoculation. First, luc activity was measured in tissue homogenates that were prepared from the local injection site at which the phage particles were injected. Luc activity in these tissue homogenates was normalized in terms of protein content and the results are shown in Fig. 5(c). Higher levels of luc expression were found in tissue homogenates prepared from animals that were injected with 3JCLI4-targeted phage, when compared with untargeted phage. The results achieved statistical significance at the day 3 time point. Similar findings were obtained when luc activity was measured by direct *in vivo* imaging of gene expression using the Xenogen IVIS system (Fig. 5d).

**Figure 6:**
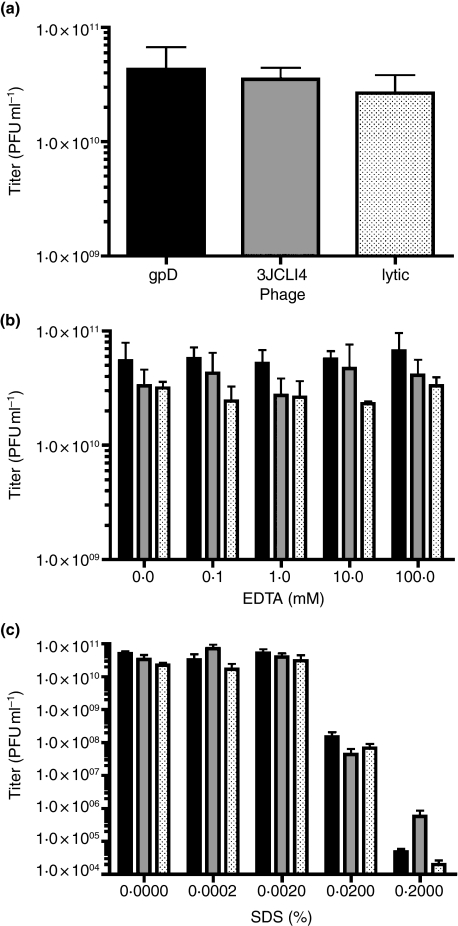
Surface display of an integrin-binding peptide (3JCLI4) does not alter the physical stability of lambda phage particles. The physical stability of various lambda phage particles was assessed using a panel of *in vitro* assays. For these experiments, the lambdaD1180 (luc) phage genome, which contains an amber termination mutation in the gpD coat protein gene, was packaged into phage particles either using an *in vitro* complementation system to express wild-type gpD (black bars) or gpD-3JCLI4 fusion protein (grey bars) on the phage surface (Zanghi *et al.* 2005) or by lytic-phase growth of the phage in *Escherichia coli* host cells containing an amber suppressor gene (‘lytic phage’, denoted by dotted bars). The physical stability of the resulting phage particles was assessed. Phages were incubated in naïve BALB/c mouse sera (diluted 1:10 in SM) at 37°C for 30 min (a), or in increasing concentrations of EDTA (b) and SDS (c), as described in the Materials and Methods section. Data shown represent mean phage titre values ± SDs. There were no statistically significant differences in the stability of phage bearing the chimeric gpD fusion protein (3JCLI4; grey bars), when compared with otherwise identical phage that displayed only wild-type gpD coat protein (gpD; black bars), except at the highest concentrations of SDS (0·02% and 0·2%). However, these SDS concentrations resulted in almost complete inactivation of phage infectivity (>99%), regardless of the coat protein composition of the phage particles.

In order to determine whether the increase in luc expression might be secondary to changes in the physical stability of phage particles that display the integrin-binding 3JCLI4 peptide on their surface, follow-up studies were conducted. These experiments assessed the stability of the 3JCLI4-targeted (luc) phage *vs* wild-type gpD (luc) and wild-type lambdaD1180 grown via a lytic method. To mimic *in vivo* conditions, phages were incubated in naïve BALB/c mouse sera at 37°C (Fig. 6a). Phages were also incubated in various concentrations of EDTA (Fig. 6b) and SDS (Fig. 6c). There were no statistically significant differences in stability of 3JCLI4-targeted (luc) phage *vs* wild-type gpD (luc), except at the highest concentrations of SDS tested (0·02% and 0·2%). However, these extreme conditions resulted in almost complete loss of phage infectivity (>99%), regardless of the coat protein composition of the phage particles. Thus, it seems unlikely that the increase in *in vivo* luc expression observed in mice receiving 3JCLI4 (luc) phage is due to an effect on phage stability.

### Phage-mediated gene transfer is modestly affected by depletion of phagocytic cells

**Figure 7:**
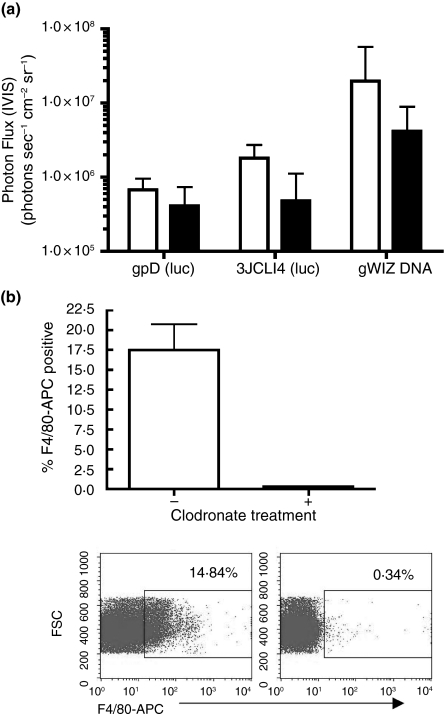
*In vivo* luciferase (luc) expression in BALB/c mice injected with wild-type or 3JCLI4-targeted phage is only modestly affected by clodronate-mediated depletion of phagocytic cells. (a) Mice (four per group) were injected with clodronate liposomes via a combined IP and ID route, to deplete both local and systemic phagocytic cells (black bars) or were left untreated (white bars). Forty-eight hours later, 1 × 10^11^ PFU of either gpD (luc) phage or 3JCLI4 (luc) phage, or 100 *μ*g of gWIZ (luc) plasmid DNA was injected ID at the tail base site, and luc expression was measured 24 h thereafter at the tail base site of injection. A decrease in *in vivo* luc gene expression in animals treated with clodronate liposomes prior to phage or DNA injection was observed, although this decrease was not statistically significant (*P* > 0·05, Student's two-tailed *t*-test). (b) After imaging, mice were killed and splenocytes were stained for F4/80 (a cell surface marker for macrophages). Mice that received clodronate liposomes had a decrease in F4/80-positive splenocytes, as measured by flow cytometric analysis. A representative staining profile for one control animal (left) and one clodronate-treated animal (right) is shown in the lower part of panel b, and mean data from all animals are shown in graphical form in the upper part of panel B (bars denote SDs).

To determine whether phage particles might be taken up via phagocytosis, as suggested by March and coworkers (Clark and March 2004; Jepson and March 2004; March *et al.* 2004), luc expression was measured following *in vivo* depletion of phagocytic cells. Briefly, clodronate-containing liposomes were administered to mice, in such a way as to deplete phagocytic cells both locally and systemically (van Rooijen and van Nieuwmegen 1984; van Rooijen *et al.* 1989; van Rooijen and van Kesteren-Hendrikx 2002). Forty-eight hours thereafter, 1 × 10^11^ PFU of either wild-type gpD (luc) phage or targeted 3JCLI4 (luc) phage, or 100 *μ*g luc-encoding plasmid DNA (gWiz) was injected ID. A moderate decrease in luc gene expression was detected in animals that had been pretreated with clodronate-containing liposomes, although this decrease did not achieve statistical significance (Fig. 7a). The efficiency of clodronate-mediated cell depletion was confirmed by immunophenotyping of splenocytes using an antibody specific for F4/80 (a cell surface marker expressed on macrophages) (Fig. 7b).

## Discussion

Bacteriophage lambda represents a promising gene transfer/vaccine delivery system. March and colleagues recently showed that unmodified lambda phage particles encoding HbsAg under the control of a CMV promoter can elicit strong humoural immune responses in mice and rabbits after booster vaccinations (Clark and March 2004; March *et al.* 2004).

In light of these findings, we wished to examine the mechanism(s) by which unmodified lamdba phage particles might transduce mammalian cells *in vivo*, and to explore ways in which this might be enhanced. We therefore developed an *in vivo* assay, which would allow us to quantify phage-mediated gene transfer in live animals, using the Xenogen IVIS system. We then used this system to address basic questions concerning phage-mediated gene transfer and immunization.

March and coworkers reported increased antibody responses following multiple IM injections of their HBsAg-encoding phage particles (Clark and March 2004; March *et al.* 2004) and speculated that this might reflect antibody-mediated opsonization of phage during subsequent immunizations, resulting in more efficient uptake by APCs (March *et al.* 2004). In keeping with this prediction, we found that the presence of pre-existing phage-specific antibodies (elicited by immunization with wild-type phage) was associated with an increase in phage-mediated reporter gene expression.

We also created modified phage particles that contained specific modifications to the major coat protein, gpD, intended to allow phage particles to more efficiently bind and transduce host APCs (Zanghi *et al.* 2005). Phage vectors that displayed a peptide known to bind to the *α*_v_*β*_3_ integrin receptor (Richards *et al.* 2003) were found to be efficiently internalized into mammalian cells that expressed this receptor, and to mediate an enhanced efficiency of gene transfer in *α*_v_*β*_3_-positive cells, *vs* otherwise identical host cells that did not express this receptor. Interestingly, there was a 100-fold increase in the efficiency of phage internalization into *α*_v_*β*_3_-positive cells *vs* control cells (Fig. 5a), but only about a threefold increase in the level of phage-mediated gene expression (Fig. 5b). This suggests that only a small fraction of the internalized phage particles were able to effectively deliver their genetic payload.

Consistent with the results of these *in vitro* experiments, *in vivo* studies suggest that targeting of phage to integrin receptors can indeed increase the efficiency of phage-mediated gene transfer following ID delivery via the tail base route, although we cannot formally rule out the possibility that the 3JCLI4–gpD coat protein modification may result in extended phage persistence at the local site of injection (hence giving rise to increased expression at the 72 h time point).

In order to try to address possible differences in the physical stability of wild type *vs* modified phage capsids, we performed a series of experimental analyses, using EDTA, SDS and serum exposure. These experiments were especially important, because our phage particles were generated using a *trans*-complementation system, in which the gpD coat protein is constitutively expressed from a bacterial plasmid, in a gpD-deficient lambda lysogen (Sternberg and Hoess 1995; Eguchi *et al.* 2001; Zanghi *et al.* 2005). It is possible that this complementation system may generate phage particles that have an inherent head instability created by this complementation system, particularly if some phage particles contain less than normal levels of gpD. Reduced levels of major coat protein are known to result in particle instability in certain lambda recombinants (Wendt and Feiss 2004), and an analogous coat protein in the dsDNA phage P4 is believed to function by preventing DNA leakage from the phage head (Dokland *et al.* 1993; Isaksen *et al.* 1993). We therefore performed a series of experiments to examine the physical stability of our gpD-complemented phage particles. These experiments showed that the physical stability of phage particles containing the wild-type gpD protein or the 3JCLI4–gpD fusion protein was essentially indistinguishable. The particles were also found to be of essentially equivalent stability to phage particles that contained an identical lambda DNA genome, but which were generated using a completely different method that did not rely upon coat protein complementation (these particles were produced by lytic-phase growth of the lambdaD1180 phage in *E. coli* host cells that were able to suppress the amber mutation in the endogenous phage gpD gene).

In order to address the mechanism of phage-mediated gene transfer, and to test whether phagocytosis was required for this process, mice were pretreated with clodronate-containing liposomes. This resulted in extensive depletion of phagocytic cells but did not significantly decrease phage-mediated gene transfer (although a nonsignificant trend towards reduced luc expression was detected). The simplest explanation for these findings is that phagocytosis is responsible for a portion, but not the majority, of phage-mediated gene transfer. It is therefore likely that other uptake mechanisms may contribute to the uptake of phage particles and expression of phage-encoded proteins. These include macropinocytosis, a process that is induced by many microbial pathogens (Swanson and Watts 1995; Amyere *et al.* 2002).

Cell types capable of performing macropinocytosis include epithelial cells and keratinocytes (Zenni *et al.* 2000; Basner-Tschakarjan *et al.* 2004), which would not be depleted by exposure to clodronate liposomes, as well as CD8+ interdigitating DC, which are known to be clodronate-resistant – unlike marginal DC and macrophages (Leenen *et al.* 1998). Further studies will be needed to determine whether macropinocytosis does indeed contribute to phage-mediated gene transfer. This might explain previous reports that a human fibroblast cell line (CCL-72 cells) can be transduced by wild-type lambda phage particles under high (4 × 10^5^) MOI conditions, leading to transcription of phage-encoded genes within the human host cells (Merril *et al.* 1971; Geier and Merril 1972). It is important to note, however, that the experiment with clodronate liposomes was performed only using nonmodified phage particles in naïve host animals. Hence, the conclusion that phage particles are taken up in large part by nonphagocytic cells may not apply to integrin-targeted phage or to phage:antibody conjugates.

Previous studies from the March group have examined immune responses to lambda phage-encoded gene products rather than phage-directed gene expression *per se* (Clark and March 2004; Jepson and March 2004; March *et al.* 2004,^2006^). These studies concluded that phage was superior to a matched plasmid vector, from the standpoint of inducing a humoural immune response to an encoded antigen driven from a CMV promoter (Clark and March 2004; Jepson and March 2004; March *et al.* 2004). In contrast, our experiments suggest that total levels of *in vivo* reporter protein expression are very roughly equivalent for naked DNA delivery *vs* lambda phage delivery, at least when following ID delivery of unmodified phage particles in naïve mice. This suggests that phage vectors may be more efficient in transducing key cell types that are necessary for efficient antigen presentation *in vivo*, when compared with naked plasmid DNA. Whether this is due to direct transduction of professional APC, such as dendritic cells [or specific, clodronate-resistant subsets of DC (Leenen *et al.* 1998; Ciavarra *et al.* 2000)], or the result of transduction of other cell types (and subsequent cross-presentation of phage-encoded antigens), remains to be determined.

In summary, our results also show that specific surface modifications of the phage particles (such as inclusion of the integrin-targeting peptide) result in increased levels of phage-mediated gene transfer. These results are compatible with a model in which phage particles may induce or enhance cellular macropinocytosis, possibly via the engagement of specific cell surface receptors or signalling pathways (Dharmawardhane *et al.* 2000). Moreover, these findings are consistent with previous studies using gpD-complemented lambda phage particles, which have shown that *in vitro* gene-transfer efficiency can be substantially increased through surface display of the HIV-1 Tat protein transduction domain (PTD) (Eguchi *et al.* 2001). This PTD is known to engage a macropinocytosis-dependent cell uptake pathway (Wadia *et al.* 2004). Parenthetically, this PTD also contains highly basic residues that may alter the electrostatic properties of the phage surface.

Overall, our work has increased the understanding of lambda-phage-mediated gene transfer and suggests new approaches that may lead to the design of second-generation phage vectors with improved gene transfer characteristics.

## Supplementary Material

The following supplementary material is available for this article online:

Figure S1. *In vivo* luciferase (luc) expression in BALB/c mice is localized to the tissue site of injection. Mice were injected ID at the tail base with 1 × 10^11^ PFU of gpD (luc) phage (a) or 1 × 10^11^ PFU of gpD (no luc) phage (b). Twenty-four hours later, luc expression was imaged at the tail base site of injection. The results are shown for a representative set of three animals.

This material is available as part of the online article from http://www.blackwell-synergy.com
